# Large Bilateral Coronary Artery Fistula: 10-year Follow-up in Clinical Treatment

**DOI:** 10.5935/abc.20180267

**Published:** 2019-02

**Authors:** Rodrigo Melo Kulchetscki, Luka David Lechinewski, Luciana Oliveira Cascaes Dourado, Whady Armindo Hueb, Luiz Antonio Machado César

**Affiliations:** 1 Instituto do Coração (InCor) do Hospital das Clínicas da Faculdade de Medicina da Universidade de São Paulo (HCFMUSP), São Paulo, SP - Brazil; 2 Hospital da Irmandade da Santa Casa de Misericórdia de Curitiba, Curitiba, PR - Brazil

**Keywords:** Arterio-Arterial Fistula/diagnosis, Coronary Angiography, Diagnostic, Imaging, Radionuclide Imaging, Coronary Vessel Anomalies, Mitral Valve Insufficiency, Myocardial Ischemia

We report on the 10-year evolution of an asymptomatic patient with a large bilateral coronary artery-pulmonary artery fistula for whom clinical treatment was chosen. Published previously,^1^ the report reinforces the need for treatment individualization in patients with moderate coronary fistulas.

## Case Report

A 59-year-old asymptomatic female patient, with a diagnosis of a large bilateral coronary-pulmonary artery fistula made in 2007 was investigated after a cardiac murmur was identified on a routine examination. At the time, conservative treatment was chosen. Cardiac auscultation showed a more audible systolic-diastolic murmur in the upper left sternal border, with a more audible component in systole. There were no other findings in the cardiological physical examination or even the overall segmental examination. The patient had no comorbidities at the time, except for a prior history of smoking (10-pack-years). During the evolution, at the annual outpatient follow-up, she had diagnoses of dyslipidemia, glucose intolerance and depression. At the last consultation, in 2017, the patient was asymptomatic. She used atenolol 25 mg/ day, metformin 850 mg/day, atorvastatin 20 mg/day and sertraline 50 mg/day.

The examinations performed after 10 years of follow-up were compared with those at the time of diagnosis. The current echocardiogram showed right coronary (RC) with 4 mm of diameter at the origin and 7 mm in the middle third; the left main coronary artery (LMCA) with 8 mm. The patient had a fistulous trajectory with tortuous flow communicating both coronaries with the pulmonary trunk, without the presence of pulmonary hyperflow. Additionally, the evolution of mitral regurgitation showed to be of an important degree. Table 1 shows the echocardiographic parameters during follow-up.

**Table 1:** Evolution of echocardiographic parameters along the years

	2007	2013	2016	2017
Left Atrium (mm)	30	37	40	38
Interventricular Septum (mm)	7	9	9	8
LV Posterior Wall (mm)	7	8	8	8
LV Diastolic Diameter (mm)	54	56	58	57
LV Systolic Diameter (mm)	37	38	39	41
LVEF (%)	59	60	60	59
Aortic Sinus (mm)	31	32	33	32
RV Systolic Function	Normal	Normal	Normal	Normal
Additional findings	Mild MR	Mild MR.Mild TR.Minimal AR.	Minimal systolic displacement of the posterior cusp towards the left atrium.Moderate MRMild degree TR.Mild PF.	Posterior cusp prolapse towards the left atrium.Important MR(eccentric jet directed to the interatrial septum).Qp/Qs ratioof 0.8.

LV: left ventricle; LVEF: left ventricular ejection fraction; RV: right ventricle; MR: mitral regurgitation; TR: tricuspid regurgitation; AR: aortic regurgitation; PF: pulmonary failure; Qp/Qs: pulmonary artery and aortic flow ratio.

Myocardial scintigraphy with dipyridamole and 99m-technetium-sestamibi showed no changes in perfusion, as well as the previous examinations performed in 2007 and 2011. The ergospirometry treadmill test (modified Balke protocol, 3.4 mph), lasting 7 minutes and 38 seconds, was maximal (109% of maximal HR), with VO_2_ peak of 22.4 mL/kg/min (87% of predicted VO_2_).

**Figure 1:**
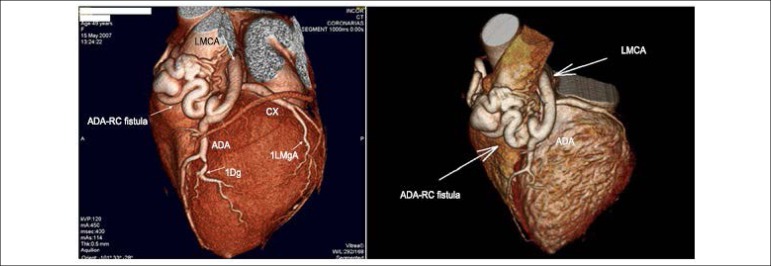
Comparative image of the coronary fistula (to the left in 2007 and to the right in 2017) - ADA: Anterior Descending Artery; LMCA: Left Main Coronary Artery; Cx: Circumflex Artery; 1Dg: First Diagonal Artery; 1LMgA: First Left Marginal Artery.

The angiotomography of the coronary arteries was performed in 2017 and the comparison with the 2007 examination can be seen in Figure 1. The finding of a systemic-pulmonary fistula persists, in the RC + ADA with the LMCA, described as the presence of a high-caliber branch emerging from the right coronary artery origin, with a tortuous trajectory, surrounding the pulmonary trunk anteriorly and communicating with the proximal third of the anterior descending artery. It shows communication with the pulmonary trunk, associated with two aneurysms along its trajectory, measuring 19x16 mm and 14x13 mm. There is no pulmonary dilation or other signs suggesting hemodynamic repercussion. Total coronary calcium score of 246 (Agatston), corresponding to the 99^th^ percentile for the age group and gender, and absence of significant coronary luminal reduction were also observed.

## Discussion

Coronary fistulas (CFs), abnormal communications between one or more coronary arteries with some cardiac or thoracic structure, usually congenital in origin,^2^ have a prevalence of 0.05% to 0.88%, depending on the diagnostic method used.^3^ They originate from one or more branches of the coronary arteries, and the pulmonary trunk is the most frequent termination of bilateral CFs.^2,4^ They may be associated with mitral regurgitation/mitral valve disease - a finding present in this case - atrial and/or ventricular septal defect, pulmonary stenosis and atresia.^5^ In the adult population, 75% are symptomatic, with chest pain and dyspnea being the most frequently complaints. Heart murmur is observed in 37% of patients at clinical examination.^5^

Patient evolution seems to be quite variable and depends on the size and hemodynamic repercussion of the CF, in addition to associated malformations. Long-term follow-up^2,4^ shows that patients can progress from being asymptomatic to symptoms of heart failure due to decreased ejection fraction, left atrial enlargement and pulmonary hypertension, and a few with coronary aneurysm, which is associated mainly with unilateral fistulas. Coronary aneurysms may favor coronary rupture and may also generate ischemia through the flow steal mechanism.^5,6^

The ideal treatment of CFs remains uncertain, especially regarding the moderate and asymptomatic cases. The conservative treatment should be considered in small, asymptomatic fistulas. The fistula spontaneous closure is rare and occurs in only 1-2% of cases.

The interventional treatment for CF closure, whether surgical or percutaneous, should be considered in large CFs and in more proximal locations, presence of symptoms, presence of other cardiovascular diseases / associated cardiac malformations, and hemodynamic repercussion (high-flow fistulas).^5,8^ However, these are not complication-free procedures.

The surgical treatment can show a high rate of periprocedural myocardial infarction and occurrence of residual tricuspid reflux.^9^ Percutaneous treatment with occlusion devices (coils used in small fistulae and Amplatzers used in large CFs)^8,10^ may also be complicated by aneurysmal dilatation and thrombosis leading to embolization and myocardial ischemia, as well as device migration ( mainly coils in large, high-flow fistulas). Situations in which occlusion is incomplete favor the occurrence of infective endocarditis and hemolysis.^5,8^

In the present case, initially described 10 years ago, of an asymptomatic moderate CF without clinical or hemodynamic repercussions, where we chose to carry out a clinical follow-up, we observed a very favorable evolution, with the patient remaining asymptomatic and with good aerobic (cardiovascular) fitness throughout the period, in the absence of myocardial ischemia and pulmonary hyperflow, with preserved ventricular function, and showing a slight increase in the RC (6 to 7 mm) and the LMCA (7 to 8 mm) diameters, in addition to a slight left chamber dilatation, the latter justified by mitral valve prolapse that developed into significant regurgitation, an association found in some cases.

As previously discussed,^1^ we emphasize that the conservative treatment is safe and should be carried out in asymptomatic patients and / or those without complications, as the one described in this case report. In symptomatic or complicated patients, however, percutaneous or surgical interventions are indicated.

This report shows, once again, the need for the individualization of management in the presence of the diagnosis of asymptomatic coronary artery fistula.
